# Incidence and Predicted Risk Factors of Pressure Ulcers in Surgical Patients: Experience at a Medical Center in Taipei, Taiwan

**DOI:** 10.1155/2014/416896

**Published:** 2014-06-26

**Authors:** Ling Fu Shaw, Pao-Chu Chang, Jung-Fen Lee, Huei-Yu Kung, Tao-Hsin Tung

**Affiliations:** ^1^Department of Nursing, Taipei Veterans General Hospital, Taipei 11220, Taiwan; ^2^Faculty of Nursing, School of Nursing, Yang-Ming University, Taipei 11220, Taiwan; ^3^Department of Medical Research and Education, Cheng Hsin General Hospital, Taipei 11220, Taiwan; ^4^Faculty of Public Health, School of Medicine, Fu Jen Catholic University, Taipei 24205, Taiwan

## Abstract

*Purpose*. To explore the context of incidence of and associated risk factors for pressure ulcers amongst the population of surgical patients. *Methods*. The initial study cohort was conducted with a total of 297 patients admitted to a teaching hospital for a surgical operation from November 14th to 27th 2006 in Taipei, Taiwan. The Braden scale, pressure ulcers record sheet, and perioperative patient outcomes free from signs and symptoms of injury related to positioning and related nursing interventions and activities were collected. *Results*. The incidence of immediate and thirty-minute-later pressure ulcers is 9.8% (29/297) and 5.1% (15/297), respectively. Using logistic regression model, the statistically significantly associated risk factors related to immediate and thirty-minute-later pressure ulcers include operation age, type of anesthesia, type of operation position, type of surgery, admission Braden score, and number of nursing intervention after adjustment for confounding factors. *Conclusion*. Admission Braden score and number of nursing intervention are well-established protected factors for the development of pressure ulcers. Our study shows that older operation age, type of anesthesia, type of operation position, and type of surgery are also associated with the development of pressure ulcers.

## 1. Introduction

Pressure ulcers are known as bed scores and decubitus ulcers and occur mainly in parts of the body that are subject to high pressure from body weight on bony prominences [1]; thus they have been defined as “an area of unrelieved pressure usually over a bony prominence leading to ischemia, cell death, and tissue necrosis” [2]. This disease often manifests negative outcomes for patients after surgeries, which may include pain, additional treatment and surgery, longer hospital stays, disfigurement or scarring, increased morbidity, and increased medical costs [3]. The development of pressure ulcer in hospitalized patients who have undergone a surgical procedure is also prompted [2–6]. In addition, pressure ulcers have been described as one of the most costly and physically debilitating complications in the 20th century [7]. Because pressure ulcers remain a major health postsurgery problem; identification of patients at risk for pressure ulcer development is imperative for implementing cost-effective, evidence-based preventive measures. Continuous risk assessment could be viewed as the continuous clinical view and judgment of the patient's pressure ulcers risk, with the goal of conducing preventive measures that meet the particular risk factor.

A multidisciplinary method is necessary in pressure prevention and treatment and a large part of the responsibility falls on nurses in this approach [5]. From the viewpoint of preventive medicine, it is important to not only be cognizant of the background morbidity of pressure ulcers regionally, but also explore the complete spectrum of demographic and biological markers which may be related to the development of pressure ulcers. Although numerous studies focusing on morbidity and risk factors of pressure ulcers have been conducted in western countries [6–9], to the best of our knowledge, however, such studies are limited—few national or local databases of surgical patients' records regarding pressure ulcers morbidity are found in Taiwan. It is essential to identify patients at risk and to plan appropriate interventions to prevent the development of pressure ulcers [5]. In order to improve the quality of care for patients with surgical operation, it is necessary for the healthcare professionals in Taiwan to acquire knowledge of the risks for pressure ulcers development and clinical risk factors and further preventive measurement pertaining to pressure ulcers is also necessary. Nurses are also responsible in the places they work for identifying patients at risk for pressure ulcers and for carrying out the pressure ulcer preventive measures [5]. This study is designed to explore the potential perioperative factors and related nursing interventions and activities then to improve the understanding of the overall pathogenesis of pressure ulcers. The purpose of this study is to explore the context of immediate and thirty-minute-later incidence of and associated risk factors for pressure ulcers amongst the population of surgical patient, as determined by the application of the subjects' study program at a fully certified medical center and teaching hospital in Taipei, Taiwan.

## 2. Methods

### 2.1. Study Design and Sample Selection

This study was conducted in a medical center in Northern Taiwan. The research sample included patients who agreed to participate, who were 18 years old or older, who were able to communicate in either Mandarin or Taiwanese, having had a first time elective surgery and a surgical procedure lasting more than 30 minutes under spinal or general anaesthesia, and who had neither existing pressure ulcers nor any traumas before surgery. The study spanned from November 14th to 27th, 2006. Patients were enrolled from the medical specialisms of cardiovascular, general surgery, chest surgery, orthopedic surgery, neurosurgery, plastic surgery, and urologic surgery. The investigation was of an observational follow-up study design, and therefore the study sample selected all patients who were listed on the surgical elective schedule during the research period. Finally, a total of 297 patients agreed to participate in the study.  Table 4 shows the study flowchart of the recruitment. Written permission was obtained from the study institution and managements that were involved. Informed consents in this study were obtained verbally from the patients prior to surgery. The surgeons and perioperative nurses of the operating room were also informed about the procedure of the study. All procedures were performed in accordance with the guidelines of ethics and adhered to the tenets of the Declaration of Helsinki. All subjects' information remained anonymous and was only used for analytical purposes.

### 2.2. Data Collection

Data were collected by using structured questionnaires including The Braden Pressure Ulcer Risk Assessment scale (a summated rating scale made up of six subscales scored from 1 to 3 or 4, for total scores that range from 6 to 23; a lower Braden scale score indicates a lower level of functioning and, therefore, a higher level of risk for pressure ulcer development) [10], pressure ulcers record sheet, perioperative patient outcomes free from signs and symptoms of injury related to positioning and related nursing interventions and activities, operation time, type of anesthesia and surgical positioning, body temperature and blood pressure, occurrence of shear power and wetness during operation, and use of heart-lung machine. Demographic characteristics such as gender, age, body mass index, personal past diseases, and nutrition were assessed preoperatively by perioperative nurse leaders. The type of anesthesia and surgical positioning, use of heart-lung machine, measurement of blood pressure during operation, and the occurrence of shear power and wetness were collected intraoperatively. Body temperature was observed immediately after surgical operation.

Pressure ulcers were defined by the National Pressure Ulcer Advisory Panel (NPUAP) and European Pressure Ulcer Advisory Panel (EPUAP) as “localized injury to the skin and/or underlying tissue usually over a bony prominence as a result of pressure or pressure in combination with shear and/or friction” [2, 11]. In this study, the perioperative nurse leaders and nurses of the operating room and postanesthesia recovery room who participated in the study were informed about the study design and received a pressure ulcer assessment evaluation tests. The occurrence of the pressure ulcer was observed both immediately after operation in the operating room and 30 minutes postoperatively in the postanesthesia recovery room. The thirty-eight nursing interventions and activities were also recorded [12]. This study selected one perioperative outcome “patient is free from signs and symptoms of injury related to positioning” and related nursing interventions from the development of an outcome-oriented perioperative nursing data set in Taiwan.” The content validation was ascertained via expert validity and the inclusion criteria were set at a CVI larger than 0.80; in addition, the CVI of above selected thirty-eight nursing intervention items was 1.0. In addition, in order to set up a consistent diagnosis of pretest and posttest pressure ulcers, the results of the test and retest reliability were examined by paired *t*-test (*t* = 11.9, *P* < 0.0001); that is, the study nurses demonstrate accuracy of the data collection for pressure ulcers.

### 2.3. Statistical Analysis

The statistical analysis was performed using SAS 9.1 (SAS Institute, Cary, NC, USA). In the univariate analysis, the *χ* ^2^-test and independent *t*-test method were adopted to assess the differences of the mean value of categorical and continuous variables, respectively. The logistic regression model was used to assess the effects of relevant factors on each type of pressure ulcer after adjustment for the covariates. Odds ratio (OR) and 95% confidence interval (CI) were used for the independent effect of associated variables. A *P* value of <0.05 was considered statistically significant.

## 3. Results

The gender specific information of the 297 study patients is shown in Table 1. The distribution of smokers (female: 6.7%, male: 28.4%, *P* < 0.0001), type of anesthesia (female: 73.3%, male: 61.1%, *P* = 0.03), type of general surgery (female: 47.4%, male: 56.2%, *P* = 0.03), operation age (female: 59.9 ± 14.5, male: 64.9 ± 15.4, *P* = 0.004), and hematocrit (female: 36.9 ± 5.5, male: 38.6 ± 6.5, *P* = 0.02) had statistical significant difference between male and female.

As Table 2 shows, there are 29 and 15 patients who were diagnosed as stage I immediate and thirty-minute-later pressure ulcer. The incidence of immediate and thirty-minute-later pressure ulcer is 9.8% (29/297) and 5.1% (15/297), respectively. Type of anesthesia (OR = 16.14, 95% CI: 2.16–120.47), type of operation position (prone versus supine, OR = 62.98, 95% CI: 16.98–233.55; lateral versus supine, OR = 14.32, 95% CI: 3.37–60.91), type of surgery (orthopedics surgery versus general surgery, OR = 5.88, 95% CI: 2.24–15.43), operation age (OR = 1.03, 95% CI: 1.00–1.06), admission Braden score (OR = 0.85, 95% CI: 0.75–0.97), and number of nursing intervention (OR = 0.95, 95% CI: 0.89–0.99) are significantly relevant to immediate pressure ulcers. In addition, the significant risk factors related to pressure ulcers of 30 minutes later included type of anesthesia (OR = 7.45, 95% CI: 1.00–57.51), type of operation position (prone versus supine, OR = 22.10, 95% CI: 5.72–85.43), type of surgery (orthopedics surgery versus general surgery, OR = 18.33, 95% CI: 2.31–145.69; cardiac surgery versus general surgery, OR = 22.00, 95% CI: 2.19–221.34), heart-lung machine used (OR = 5.27, 95% CI: 1.02–27.34), operation age (OR = 1.04, 95% CI: 1.00–1.09), admission Braden score (OR = 0.84, 95% CI: 0.71–0.98), and number of nursing intervention (OR = 0.94, 95% CI: 0.90–0.98).

The effects of independent associated factors of each type of pressure ulcers are examined by the multiple logistic regression model in Table 3. The statistically significantly associated risk factors related to immediate pressure ulcer include operation age (OR = 1.03, 95% CI: 1.00–1.08), type of anesthesia (general anesthesia) (yes versus no, OR = 17.06, 95% CI: 2.09–49.43), type of operation position (nonsupine versus supine, OR = 32.26, 95% CI: 4.48–48.79), type of surgery (orthopedics surgery versus general surgery, OR = 3.33, 95% CI: 1.05–10.61), admission Braden score (OR = 0.95, 95% CI: 0.91–0.99), and number of nursing intervention (OR = 0.94, 95% CI: 0.90–0.98). Operation age (OR = 1.06, 95% CI: 1.00–1.12), type of operation position (nonsupine versus supine, OR = 18.18, 95% CI: 1.32–52.63), type of surgery (orthopedics surgery versus general surgery, OR = 9.29, 95% CI: 1.05–28.50; cardiac surgery versus general surgery, OR = 22.60, 95% CI: 1.20–43.85), and number of nursing intervention (OR = 0.95, 95% CI: 0.91–0.99) are independently significant relevant to pressure ulcers of 30 minutes later after adjustment for confounding factors.

## 4. Discussion

### 4.1. Morbidity of Pressure Ulcer

Previous studies focused on nonblanchable erythema as the early identification of pressure ulcer and investigated the factors for developing into more severe pressure ulcers [13]. In this study, the reasons for evaluating pressure ulcer at two points of time were because blanchable erythema is the first clinical sign of pressure ulcer development, especially over a bony prominent area after surgery. Incidence of blanchable erythema and deterioration to pressure ulcer were reported on surgical patients [13]. Postoperative patients routinely stayed in the postanesthesia recovery room for at least two hours unmoved; blanchable erythema could worsen to pressure ulcer of either stage I or II. Detecting the blanchable erythema is expected to provide appropriate care to prevent pressure ulcer.

Early detection of pressure ulcer has been emphasized because it could prevent skin alteration from progressing to skin loss. Patients are exposed to complications during surgical operations for reasons associated with the surgical position and for many other causes. It is known that pressure ulcers are lesions caused by unrelieved pressure that results in damage to the underlying tissue. This disorder is a health problem that brings both high material and emotional losses to patients [5]. Generally, these are the results of soft tissue compression between a bony prominence and an external surface for a prolonged period of time [14]. Knowledge of pressure ulcer epidemiology is therefore crucial in managing this disorder, not only for planning preventive programs, but also for the identification of the best therapeutic strategy. The incidence of pressure ulcer amongst different test populations appears to vary, differing among different studies conducted in different countries. In this study, the incidence of immediate and thirty-minute-later pressure ulcers is 9.8% and 5.1%, respectively. Incidence rates of pressure ulcers as low as 0.4% to as high as 38% have been reported in the inpatient department while prevalence has been reported as 3.5% to 69% [2, 8, 9, 15–17]. In long-term care facilities, the reported incidence is between 2.2% and 23.9%, while in home care setting the incidence varies from 0 to 17% [15]. Many trials have chosen not to include them since they are difficult to be reliably detected although stage 1 ulcers are frequently encountered [18]. Further well-designed epidemiological investigations of pressure ulcers in various settings are still required. In addition, not surprisingly, the hospital stay is longer in pressure ulcer patients with both excess likelihood of nosocomial, renal infections and the hospital readmission rate. A previous study based on the hospital billing codes also revealed an increase in the number of hospital stays involving pressure ulcers by nearly 80% [19]. This implies that pressure ulcers result in an exponential increase in the healthcare burden and financial requirement for these patients.

### 4.2. Implications as Regards Associated Risk Factors for Pressure Ulcer

In Taiwan, one study on elective surgical patient revealed that the incidence of the perioperative pressure ulcer in surgical patients was 7.0%, and the significant factors associated with pressure ulcer development were age, preoperative chronic cerebral arterial disease, preoperative BMI, total protein level, albumin level, Braden scale scores, operative time, body temperature, and intraoperative blood pressure [12]. Previous study also indicated that preoperatively all patients carry a risk for pressure ulcers, that risk postoperatively and that it is necessary to use a measure of risk to identify patients' risk for surgery-related pressure ulcer [5, 20]. In this study, none of the preoperative physical condition (i.e., past disease, hemoglobin, hematocrit, and smoking habit), nutrition (i.e., BMI and albumin), and intraoperative status (time of the operation, total time of diastolic blood pressure less than 60 mmHg, application of heart-lung machine, body temperature after procedure, and shear power and wetness) variables measured emerged as statistically significant risk factors for pressure ulcer development. It is possible that the control of the patient physical condition, such as blood pressure and body temperature, was satisfactory and did not affect the development of the pressure ulcer. And this also may be related to adequate preventive nursing interventions performed perioperatively.

The estimated incidence of pressure ulcers increased with operation age in this study. Such a finding is consistent with results of other studies conducted elsewhere [21]. It means that the long-term exposure to many other risk factors among elder persons may also account for the increased probability of developing pressure ulcers. In addition, the reduced risks for pressure ulcers found in relation to admission Braden score increased. The Braden Pressure Ulcer Risk Assessment scale was developed by Barbara Braden in 1987 and there have been many studies in the US and UK which have shown its validity and reliability [1, 5, 21]. In the determination of risk preoperatively and postoperatively with the Braden Pressure Ulcer Risk Assessment scale that was used in this study and in the determination of areas at risk [5], it could offer detailed clues for planning appropriate patient interventions for pressure ulcers.

Our results also support the hypothesis that the number of nursing interventions is at lower risk for development of pressure ulcers. The result of the study will improve perioperative nursing and provide the nursing administrators with the effects of the clinical perioperative nursing for the pressure ulcers prevention. A multidisciplinary approach is essential in prevention of pressure ulcers and a large part of the responsibility falls on nurses in this approach. Nursing staff are responsible in the institute they work in for identifying patients at risk for pressure ulcers and carrying out the preventive measures [5]. “Prevention is better than cure” is best emphasized in the case of pressure ulcers. This condition is absolutely preventable with care, compassion, and dedication towards the care of patients. Prevention is directed towards taking care of the extrinsic and intrinsic factors [2].

The variables including position, general anesthesia, and type of surgery had a statistically significant association with incident pressure ulcers in this study. Evidence was found that the chance of a patient who used general anesthesia to present pressure ulcers is 4.8 times greater than that who used local anesthesia (*P* = 0.024). It is certain that this correlation is also associated with surgery duration and size, as longer surgeries usually make use of general anesthesia [22]. It points at general anesthesia as a factor predisposing the occurrence of pressure ulcers due to immobilization and absence of skin sensitivity, in addition to changes in blood pressure, tissue perfusion, the patient's response to pain, and the oxygen and carbon dioxide exchange [3, 22]. In addition, it should be noted that neurosurgeries in the ventral position include spinal surgeries, and this could have determined the higher pressure ulcers incidence observed [22]. This hypothesis is also supported by a study that found a higher pressure ulcers incidence in patients submitted to spinal surgeries [23].

### 4.3. Methodological Considerations

Although using a follow-up study design could clarify the temporal relationship of potential risk factors for the development of pressure ulcer, there are some drawbacks in this study. A major limitation was the potential self-selection bias due to the hospital-based study design; it is not entirely representative of the whole general population. Secondly, a logistic regression of a binary response variable (*Y*) on a binary independent variable (*X*) with a sample size of 297 observations (of which 60% are in the group *X* = 0 and 40% are in the group *X* = 1) achieves 74% power at a 0.05 significance level to detect a change in Prob (*Y* = 1) from the baseline value of 0.097 to 0.212. This change corresponds to an odds ratio of 2.500. An adjustment was made since a multiple regression of the independent variable of interest on the other independent variables in the logistic regression obtained an *R*-squared of 0.100 in this study [24]. Although the identifications of pressure ulcers based on clinician-researchers directly examined patients meant estimates are accurate, the sample sizes tend to be relatively small and involve only a single facility, making generalizability uncertain, such that the type of anesthesia had broad confidence interval possibly due to the small number of local anesthesia. Thirdly, we only observed immediate and thirty-minute-later pressure ulcers. There is a possibility that they may have been affected by postoperative risk factors because some of the pressure ulcers were seen after the third postoperative day [5]. The true incidence of pressure ulcers would be underestimated. Further long-term studies should be conducted with a larger sample size to explore the morbidity and consequences of pressure ulcers and plausible biological mechanisms underlying its development.

## 5. Conclusion

In conclusion, admission Braden score and number of nursing intervention are well-established protected factor for the development of pressure ulcers. Our study shows that older operation age, type of anesthesia, type of operation position, and type of surgery are also associated with the development of pressure ulcers.

## Figures and Tables

**Table 1:** The gender specific information of study patients (*n* = 297).

Variables	Female (*n* = 135) Number (%) or mean ± SD	Male (*n* = 162) Number (%) or mean ± SD	Total (*n* = 297) Number (%) or mean ± SD	*P* value for *χ* ^2^-test or *t*-test
*Categorical variables *				
Past disease				
Yes	94 (69.6)	122 (75.3)	216 (72.7)	0.27
No	41 (30.4)	40 (24.7)	81 (27.3)	
Smoking				
Yes	9 (6.7)	46 (28.4)	55 (18.6)	<0.0001
No	126 (93.3)	116 (71.6)	241 (81.4)	
Type of anesthesia (general anesthesia)				
Yes	99 (73.3)	99 (61.1)	198 (66.7)	0.03
No	36 (26.7)	63 (38.9)	99 (33.3)	
Type of operation position				
Supine	88 (65.2)	94 (58.0)	182 (61.3)	0.17
Prone	18 (13.3)	19 (11.7)	37 (12.5)	
Lithotomy	12 (8.9)	29 (17.9)	41 (13.8)	
Lateral	13 (9.6)	18 (11.1)	31 (10.4)	
Others	4 (3.0)	2 (1.2)	6 (2.0)	
Type of surgery				
General surgery	64 (47.4)	91 (56.2)	155 (52.2)	0.03
Neurosurgery	13 (9.6)	11 (6.8)	24 (8.1)	
Orthopedics surgery	52 (38.5)	42 (25.9)	94 (31.7)	
Cardiac surgery	6 (4.4)	18 (11.1)	24 (8.1)	
Warmer used				
Yes	107 (79.3)	135 (83.3)	242 (81.5)	0.37
No	28 (20.7)	27 (16.7)	55 (18.5)	
Shear				
Yes	67 (49.6)	80 (49.4)	147 (49.5)	0.97
No	68 (50.4)	82 (50.6)	150 (50.5)	
Wet				
Yes	4 (3.0)	12 (7.4)	16 (5.4)	0.09
No	131 (97.0)	150 (92.6)	281 (94.6)	
Heart-lung machine used				
Yes	4 (3.0)	6 (3.7)	10 (3.4)	0.72
No	131 (97.0)	156 (96.3)	287 (96.6)	
Diastolic blood pressure less than 60 mmHg during operation				
Yes	104 (77.0)	111 (68.5)	215 (72.4)	0.10
No	31 (23.0)	51 (31.5)	82 (27.6)	
*Continuous variables *				
Operation age (yrs)	59.9 ± 14.5	64.9 ± 15.4	62.6 ± 15.2	0.004
Body mass index (Kg/m^2^)	24.8 ± 4.4	25.0 ± 3.6	24.9 ± 4.0	0.76
Hemoglobin (g/dL)	13.2 ± 6.8	13.7 ± 5.3	13.4 ± 6.0	0.48
Hematocrit (%)	36.9 ± 5.5	38.6 ± 6.5	37.8 ± 6.1	0.02
Admission Braden score	21.7 ± 2.4	21.6 ± 2.3	21.7 ± 2.3	0.72
Time of operation (min)	197.4 ± 111.5	210.8 ± 145.6	204.7 ± 131.1	0.38
Number of nursing intervention	35.9 ± 8.2	36.4 ± 7.9	36.2 ± 8.0	0.65
Ear temperature after operation (°C)	35.9 ± 0.8	35.9 ± 0.9	35.9 ± 0.9	0.57
Total time of diastolic blood pressure less than 60 mmHg (min)	83.8 ± 94.4	71.1 ± 100.5	76.9 ± 97.8	0.27

**Table 2:** Univariate analysis for comparison of characteristics in pressure ulcers among study population (*n* = 297).

Types of pressure ulcers						
	Immediately			30 minutes later		
	Yes (*n* = 29)	No (*n* = 268)	OR (95% CI)	Yes (*n* = 15)	No (*n* = 282)	OR (95% CI)
*Categorical variables *						
Gender						
Female	13	122	0.97	5	130	0.59
Male	16	146	(0.45–2.10)	10	152	(0.20–1.75)
Past disease						
Yes	21	195	0.98	11	205	1.03
No	8	73	(0.42–2.32)	4	77	(0.32–3.34)
Smoking						
Yes	4	51	0.68	3	52	1.11
No	25	217	(0.23–2.04)	12	230	(0.30–4.06)
Type of anesthesia (general anesthesia)						
Yes	28	170	16.14	14	184	7.45
No	1	98	(2.16–120.47)	1	98	(1.00–57.51)
Type of operation position						
Supine	3	179	1.00	3	179	1.00
Prone	19	18	62.98 (16.98–233.55)	10	27	22.10 (5.72–85.43)
Lithotomy	1	40	1.49 (0.15–14.72)	0	41	—
Lateral	6	25	14.32 (3.37–60.91)	2	29	4.11 (0.66–25.70)
Others	0	6	—	0	6	—
Type of surgery						
General surgery	6	149	1.00	1	154	1.00
Neurosurgery	2	22	2.25 (0.43–11.89)	1	23	6.70 (0.40–110.79)
Orthopedics surgery	18	76	5.88 (2.24–15.43)	10	84	18.33 (2.31–145.69)
Cardiac surgery	3	21	3.55 (0.82–15.26)	3	21	22.00 (2.19–221.34)
Warmer used						
Yes	28	214	7.06	15	227	—
No	1	54	(0.94–53.04)	0	55	
Shear						
Yes	12	135	0.70	6	141	0.67
No	17	133	(0.32–1.51)	9	141	(0.23–1.92)
Wet						
Yes	1	15	0.60	0	16	—
No	28	253	(0.08–4.73)	15	266	
Heart-lung machine used						
Yes	2	8	2.41	2	8	5.27
No	27	260	(0.49–11.92)	13	274	(1.02–27.34)
Diastolic blood pressure less than 60 mmHg during operation						
Yes	24	191	1.94	12	203	1.56
No	5	77	(0.71–5.26)	3	79	(0.43–5.66)
*Continuous variables *						
Age (yrs)	—	1.03 (1.00–1.06)		—	1.04 (1.00–1.09)	
Body mass index (Kg/m^2^)	—	1.03 (0.93–1.13)		—	1.00 (0.87–1.14)	
Hemoglobin (g/dL)	—	0.99 (0.92–1.07)		—	0.99 (0.89–1.10)	
Hematocrit (%)	—	0.99 (0.93–1.05)		—	1.04 (0.95–1.14)	
Admission Braden score	—	0.85 (0.75–0.97)		—	0.84 (0.71–0.98)	
Time of operation (min)	—	1.00 (0.98–1.02)		—	1.00 (0.99–1.01)	
Number of nursing intervention	—	0.95 (0.89–0.99)		—	0.94 (0.90–0.98)	
Ear temperature after operation	—	0.93 (0.60–1.44)		—	0.77 (0.43–1.38)	
Total time of diastolic blood pressure less than 60 mmHg (min)	—	1.00 (0.99–1.01)		—	1.00 (0.99–1.02)	

**Table 3:** Multivariate analysis using logistic regression model of risk factors associated with the pressure ulcers among study population (*n* = 297).

Variable	Types of pressure ulcers (yes versus no)			
	Immediately		30 minutes later	
	OR	(95% CI)	OR	(95% CI)
Gender (female versus male)	0.98	0.39–2.61	0.65	0.18–2.27
Operation age (yrs)	1.03	1.00–1.08	1.06	1.00–1.12
Type of anesthesia (general anesthesia) (yes versus no)	17.06	2.09–49.43	5.29	0.56–46.71
Type of operation position (nonsupine versus supine)	32.26	4.48–48.79	18.18	1.32–52.63
Type of surgery				
General surgery	1.00	—	1.00	—
Neurosurgery	1.29	0.20–8.58	5.57	0.28–19.69
Orthopedics surgery	3.33	1.05–10.61	9.29	1.05–28.50
Cardiac surgery	6.98	0.72–39.88	22.60	1.20–43.85
Heart-lung machine used (yes versus no)	5.24	0.51–43.55	7.58	0.51–49.28
Admission Braden score	0.95	0.91–0.99	0.93	0.80–1.09
Number of nursing intervention	0.94	0.90–0.98	0.95	0.91–0.99
The Hosmer-Lemeshow test	χ_(8)_^2^ = 3.79, *P* = 0.88		χ_(8)_^2^ = 4.17, *P* = 0.84	
c-statistics	0.914		0.917	

**Table 4:** The study flowchart of the recruitment.

Preoperative *N* = 297			Intraoperative *N* = 297		Postoperative *N* = 297
The day before operation	Operation day when arrived in holding area		Operation day during procedure		Operation day in postanesthesia recovery room
Selected patients from next day's elective surgical schedule included patients who (1) agreed to participate, (2) have a first time elective surgery, (3) have procedure lasting more than 30 minutes, (4) are under spinal or general anesthesia, and (5) have no existing pressure ulcers nor any traumas before surgery	Assessment of (1) the Braden scale, and (2) patient demographic characteristics and health status	$\overset{\;}{\to }$	Assessment of (1) pressure ulcers record sheet, (2) related nursing interventions and activities, and (3) operation related data: operation time, type of anesthesia, positioning, body temperature, and so forth	$\overset{\;}{\to }$	Assessment of (1) pressure ulcers record sheet and (2) related evaluation to the nursing interventions and activities
